# Characterization of Latency Sources in a MicroPython-Based ESP32 Edge–Cloud Sensor Network

**DOI:** 10.3390/s26175555

**Published:** 2026-09-01

**Authors:** Katarzyna Smelcerz

**Affiliations:** AGH University of Krakow, al. Mickiewicza 30, 30-059 Krakow, Poland; ksmelcerz@agh.edu.pl

**Keywords:** edge computing, Internet of Things, distributed sensor networks, ESP32, MicroPython, ESP-NOW, MQTT, latency decomposition, gateway processing, time synchronization

## Abstract

This paper presents the design and experimental characterization of a distributed ESP32/MicroPython edge–cloud sensing system with packet-level latency decomposition. Sensor nodes transmit periodic telemetry to an ESP32 gateway over ESP-NOW; the gateway appends reception and MQTT-publication timestamps and forwards records through a local Mosquitto bridge v2.1.2, EMQX Cloud v5, Telegraf v1.36.0, and InfluxDB Cloud Serverless (Storage Engine Version 3). A three-probe two-way gateway-referenced synchronization procedure provides corrected sender timestamps while exposing an interval-based synchronization-uncertainty diagnostic. The bridge-assisted campaign comprised three independent 30 min repetitions with one, three, and five active nodes. Across runs, mean gateway-referenced node-to-gateway latency was 23.17 ± 0.13 ms, 24.13 ± 0.12 ms, and 24.84 ± 0.47 ms, respectively; the corresponding p95 values were 28 ms, 33 ms, and 37–38 ms. Mean gateway-processing latency remained nearly unchanged at 13.31–13.46 ms. Exact full-run database-visible PDR was 100% in all one-node runs, 99.28–99.88% in the three-node runs, and 96.75–97.05% in the five-node runs. Independent GPIO/oscilloscope validation showed a reproducible positive software-to-hardware difference of 12.132 ± 1.819 ms across run means, so the local metric is interpreted as a gateway-referenced application-level delivery metric rather than unbiased physical one-way radio latency. Relative to aggregate end-to-end reporting, the instrumentation separates local, gateway, and downstream ingestion contributions rather than claiming a universally faster transport method. Quantitative performance and scaling claims are confined to the evaluated bridge-assisted configuration and controlled indoor periodic workload.

## 1. Introduction

Distributed IoT sensing systems increasingly rely on low-cost microcontrollers to acquire data close to the physical environment and forward it to cloud services for storage, monitoring, and analysis [1,2,3]. In such systems, the gateway is often presented as a simple bridge between local devices and cloud infrastructure. In practice, however, the gateway firmware may become an important source of latency, jitter, and packet loss, especially when implemented on resource-constrained hardware using cooperative multitasking, TLS-secured MQTT communication, and local buffering.

This problem is particularly relevant for MicroPython v1.24.0-based ESP32 platforms. JSON handling, and SSL/TLS support, but it also exposes the application to implementation-level constraints: a single blocking call can stall the event loop, synchronous flash writes can compete with packet reception and network forwarding, and memory pressure during TLS handshakes may affect reconnection behavior. This development trade-off is consistent with ESP32 language-performance studies, where MicroPython is reported to provide a simpler development model but substantially lower execution performance and garbage-collector-related unpredictability than compiled alternatives such as C/C++, Rust, or TinyGo [4]. These effects are rarely quantified explicitly in application-oriented IoT papers, where communication performance is often reported as an aggregate end-to-end metric or where the gateway is treated as a transparent relay [5,6,7,8,9].

The motivation of this work is therefore not to propose another generic IoT transport protocol, but to address a measurement gap that remains when an MCU-class gateway is treated as a transparent relay. Aggregate end-to-end measurements cannot distinguish local wireless delay from cooperative-scheduler delay, gateway queueing/publication effects, or backend-ingestion delay; moreover, software clock alignment can itself distort a one-way latency estimate. The aim of this work is therefore not only to present an ESP32-based environmental monitoring demonstrator, but primarily to experimentally characterize where latency is introduced in a MicroPython edge–cloud pipeline. The proposed testbed consists of multiple ESP32 sensor nodes, a central ESP32 gateway, a local MQTT bridge, a Raspberry Pi-based Telegraf service, and an MQTT–InfluxDB backend. Each telemetry packet carries sender-side metadata and is augmented at the gateway with reception and MQTT publication timestamps, enabling the measured delay to be decomposed into node-to-gateway, gateway-processing, and backend-forwarding components. The packet-delivery metric used in the evaluation is therefore intentionally database-visible: it describes continuity of the stored telemetry stream rather than the reliability of the local radio link alone.

During development, several gateway-side firmware mechanisms were identified as potentially harmful to latency observability and delivery reliability: a fixed one-second sleep in the publish loop, synchronous CSV writes executed inside the cooperative scheduling context, limited queue capacity, and blocking MQTT reconnection behavior. These mechanisms were removed or reduced in the revised gateway configuration by introducing bounded batch publishing, short idle sleeps, disabled synchronous local logging, a single reconnection attempt without blocking back-off delays, explicit MQTT client reset, and packet re-queuing after failed publication.

The main contributions of this paper are as follows: (i) a real ESP32/MicroPython edge–cloud testbed with packet-level timing instrumentation; (ii) a three-probe two-way gateway-referenced synchronization procedure suitable for resource-constrained nodes without direct Internet time access, with an explicit admissible offset-interval diagnostic; (iii) independent GPIO/oscilloscope validation that constrains the interpretation of the software-derived node-to-gateway metric; (iv) three independent 30 min bridge-assisted repetitions for each of one, three, and five active nodes, using a common workload and jitter policy; and (v) stage-specific reliability and gateway diagnostics including sender TX counts, gateway receive/publish counts, queue occupancy, publication failures/requeues, and free-memory reporting. Quantitative performance and scaling claims are deliberately confined to the evaluated bridge-assisted configuration. Temperature, humidity, and light sensors are used as representative environmental inputs; the primary subject of the study is the communication, timestamping, and firmware architecture, not the calibration of the sensing elements.

### Related Work

The performance of MQTT-based Internet of Things (IoT) systems has been extensively investigated from several perspectives, including broker scalability, quality-of-service (QoS) behavior, cloud deployment, protocol interoperability, local wireless communication, and edge-computing architectures. However, most existing studies evaluate MQTT or local wireless performance at the protocol, broker, middleware, link, or end-to-end communication level. Much less attention has been given to the internal latency structure of resource-constrained embedded gateways, especially when the gateway is implemented on ESP32-class hardware using MicroPython.

Several studies focus on MQTT protocol performance and broker-level benchmarking. Borsatti et al. analyzed MQTT communication across IoT, access, edge, and cloud domains, measuring end-to-end delivery latency between publishers and subscribers under different QoS levels and deployment placements [10]. Bender et al. compared open-source MQTT implementations, including Mosquitto, HiveMQ, EMQX, VerneMQ, MQTT.js, and Paho, evaluating interoperability, resource consumption, latency, and security aspects [11]. Mishra et al. performed stress testing of six MQTT brokers and analyzed their behavior in terms of CPU usage, message rate, and average latency in local and cloud-based environments [12]. More recently, Gaspar et al. investigated the practical limits of Mosquitto-based MQTT communication under varying QoS levels, payload sizes, publishing rates, client populations, publisher queue sizes, broker resources, and TCP packet aggregation strategies [13]. These works provide important insight into MQTT scalability and broker behavior, but they do not isolate latency contributions introduced by embedded gateway firmware, local queue management, or node-to-gateway communication.

Other studies have examined gateway, cloud, and cloud–edge placement in MQTT communication. Kenitar et al. evaluated MQTT latency over different gateways using an industrial setup involving a Groov Edge publisher, Node-RED, a Siemens IoT2040 fog broker, and cloud brokers [14]. Their results indicate that fog-level broker placement can reduce and stabilize MQTT latency compared with cloud placement, although the number of network hops alone does not fully determine the observed delay. Similarly, Ansyah et al. compared MQTT broker performance across AWS, Microsoft Azure, and Google Cloud Platform using Mosquitto, JMeter, and MQTTLoader, considering throughput, latency, number of subscribers, and QoS levels [15]. Tseng et al. further showed that cloud–edge MQTT messaging can suffer from size-dependent latency penalties under transient local network congestion and from broker-side memory pressure when large message copies accumulate in the cloud broker [8]. These studies confirm that broker placement, cloud infrastructure, congestion, and message scheduling influence MQTT performance, but the gateway is still treated mainly as a communication endpoint, broker-side element, or network placement problem rather than as a resource-constrained processing stage whose internal firmware behavior is analyzed separately.

A related group of works compares MQTT with other publish/subscribe or IoT communication protocols. Kang et al. evaluated DDS, MQTT, and ZeroMQ under representative IoT traffic conditions, showing that middleware performance depends strongly on load conditions and QoS configuration [16]. Mishra and Guru compared MQTT, CoAP, and HTTP in NS-3 simulations using multiple metrics, including latency, jitter, throughput, energy usage, packet delivery ratio, and network footprint [17]. Adriansyah et al. conducted a systematic literature review comparing MQTT and HTTP on ESP32-based IoT devices, reporting that MQTT generally provides lower latency, lower overhead, and better energy efficiency than HTTP in resource-constrained deployments [18]. These protocol-comparison studies support the selection of MQTT for constrained IoT systems, but they do not address how latency is distributed inside a complete ESP32 gateway-to-cloud pipeline.

The local node-to-gateway segment is also relevant to the present work. Eridani et al. compared ESP-NOW, Wi-Fi, and Bluetooth using ESP32-class devices and considered range, transmission speed, latency, energy usage, and barrier resistance [19]. Becker et al. investigated ESP-NOW performance in controlled and outdoor environments, with particular emphasis on packet delivery ratio and delay distributions [20]. These studies are important because they show that low-overhead 2.4 GHz local communication may provide favorable latency and energy properties for ESP32-based deployments. At the same time, they evaluate the local link itself, whereas the present work investigates how local-link behavior, timestamping uncertainty, gateway scheduling, MQTT forwarding, and backend ingestion appear together in a complete edge–cloud measurement pipeline.

ESP32-based edge-computing and gateway platforms form another important reference point. Gaspar et al. described MicroPython as a rapid-prototyping platform for IoT and Industry 4.0 applications, emphasizing portability, modularity, and hardware-level extensibility [21]. Serepas et al. presented a lightweight ESP32-based embedded IoT gateway for smart-home applications using MQTT over TLS, REST APIs, local control, and on-site data storage [9]. Their evaluation extends to up to 32 controlled devices and reports both command-success rates and gateway RAM use, providing useful evidence on resource-aware MCU-gateway scalability; however, the principal outcome is control reliability and resource utilization rather than packet-level decomposition of the communication path. Chang et al. developed an ESP32-CAM edge-computing system for object detection, combining local processing with cloud-side YOLOv8 verification and MQTT-based communication through local and remote brokers [22]. That work quantifies MQTT transmission time and the effect of edge/cloud placement, but evaluates a single edge node and identifies multi-node coordination and latency as future work. Thus, these studies support the feasibility of ESP32-class edge/gateway functions while leaving the internal timing structure of a multi-node embedded gateway largely unresolved.

A particularly relevant issue is the influence of the gateway software runtime and scheduling model. Plauska et al. compared C/C++, MicroPython, Rust, and TinyGo on ESP32 and found substantially lower execution performance for MicroPython in computational kernels, while also emphasizing its simpler development model [4]. Their benchmarks establish a language-runtime performance gap, but do not determine how that gap translates into wireless reception, cooperative scheduling, queue handling, or MQTT forwarding. Branch and Weinstock provide a closer gateway-level comparison: they implemented the same LoRa–MQTT gateway on ESP32 in C++ and in the interpreted Elixir/AtomVM environment and observed markedly lower delivery efficiency and higher latency for the interpreted implementation as offered load increased [23]. Because their radio technology, runtime, workload, and timing definition differ from those used here, their numerical results are not a direct benchmark for MicroPython; nevertheless, they demonstrate that software-runtime choice can become a first-order gateway-performance factor under load.

An alternative design direction is to use an RTOS to isolate time-critical reception from network forwarding. Ngo et al. implemented a multi-node ESP32 gateway in FreeRTOS and explicitly separated LoRa/TDMA communication from Wi-Fi/TCP/IP/MQTT processing across cores and task priorities [24]. This architecture targets deterministic execution and reported bounded timing behavior in a three-node deployment. In contrast, the present system deliberately uses a cooperative MicroPython/uasyncio event loop in which reception, queue processing, and publication share a scheduling context. The comparison is therefore architectural rather than numerical: RTOS-based task isolation offers stronger timing control, whereas the present study characterizes the latency and failure modes that emerge in a lightweight interpreted gateway without claiming equivalence to a FreeRTOS implementation.

Recent resource-aware edge–cloud studies also emphasize that the placement of computation and data aggregation can affect network load and latency. Ali et al. proposed an edge–cloud traffic-prediction framework in which redundant sensor information is aggregated at the edge and only relevant data are forwarded to the remote cloud, illustrating the broader role of edge processing in reducing communication overhead and supporting latency-sensitive applications [25]. Their study, however, focuses on resource-aware traffic prediction and data aggregation rather than packet-level communication timing or embedded-gateway latency decomposition.

Domain-specific monitoring systems provide additional context for the application space of the present work. ESP32/MicroPython platforms have been used for IoT warehouse monitoring with environmental and gas sensors [26], as well as for MQTT-based background-radiation monitoring with Node-RED dashboards [27]. A recent edge–cloud vertical-farming platform based on ESP32-S3/CAM nodes, C++/FreeRTOS firmware, buffering, and cloud microservices reported a 95th-percentile sensor-to-dashboard latency below 180 ms and no message loss during deliberate Wi-Fi outages [28]. These systems demonstrate the practical relevance of low-cost ESP32 monitoring platforms, but they primarily evaluate application functionality, edge data reduction, or system-level responsiveness rather than decomposing node-to-gateway, gateway-processing, and backend-forwarding latency in a MicroPython gateway.

Latency-sensitive and time-critical MQTT variants have also been proposed. Patti et al. introduced PrioMQTT, a prioritized MQTT-compatible protocol that uses UDP and message prioritization to reduce round-trip time for time-critical industrial traffic [29]. Testa et al. extended this direction by investigating the integration of PrioMQTT with Time-Sensitive Networking (TSN), aiming to provide bounded delays for flows with different real-time requirements [30]. Chauhan and Gore proposed SMQTT, a lightweight clock synchronization algorithm that leverages the MQTT publish/subscribe architecture to improve synchronization accuracy of resource-constrained IIoT devices under degraded network conditions [31]. These studies are relevant because they show that MQTT-based systems are increasingly considered for latency-sensitive industrial applications. Nevertheless, they primarily modify protocol behavior, synchronization mechanisms, or network scheduling, whereas the present work focuses on standard MQTT communication and experimentally characterizes latency sources in the gateway software path.

Time synchronization is a separate but closely related problem. Yiğitler et al. reviewed time-synchronization methods for IoT deployments and emphasized that heterogeneous WAN/LAN/PAN deployments often rely on gateways, which can introduce processing and translation overhead in applications that require interaction with the physical world [32]. Mani et al. showed that IoT prototyping platforms may exhibit clock drift on the order of seconds over relatively short periods and that standard SNTP- or MQTT-based approaches can be insufficient under noisy conditions [33]. These findings motivate the timestamping strategy used in the present work: sensor nodes remain lightweight and do not contact NTP directly, while a two-way local exchange with the gateway estimates both synchronization delay and clock offset so that node timestamps can be interpreted relative to the gateway clock.

Interoperability and security aspects of MQTT-based deployments have also been studied. Innamorati et al. proposed a broker-based extension enabling bidirectional interoperability between MQTT and CoAP while maintaining low latency and limited CPU and memory overhead [34]. Gentile et al. analyzed MQTT-based distributed measurement systems using constrained hardware, local MQTT brokers, TLS tunnels, SSL tunnels, OpenWrt routers, and Raspberry Pi devices, evaluating the influence of cipher suites, TLS versions, MQTT versions, and QoS levels on throughput-related metrics [35]. More recently, Amirkhanova et al. instrumented the packet-preparation path of an ESP32 MQTT client and decomposed its processing cost into JSON serialization, AES-GCM encryption/authentication, and Base64 encoding, reporting a mean preparation latency of 41.754 ms [36]. This result is particularly relevant methodologically because it demonstrates that tens of milliseconds can arise inside the embedded software path before network delivery. Their system, however, uses a single C++ ESP32 publisher and focuses on application-layer security overhead rather than multi-node reception, cooperative gateway scheduling, queueing, or gateway-to-backend latency decomposition. Together, these works show that security, serialization, and protocol-processing choices can materially affect embedded timing, while leaving the internal timing structure of a MicroPython multi-node gateway insufficiently characterized.

Table 1 summarizes the main research streams relevant to this manuscript and positions the contribution of the present work with respect to existing MQTT, gateway, protocol-comparison, ESP-NOW, time-synchronization, and ESP32-based edge-computing literature.

The analysis of the related literature reveals that existing studies provide strong evidence on MQTT broker behavior, QoS effects, cloud placement, protocol comparison, security overhead, local ESP-NOW communication, IoT time synchronization, ESP32 gateway feasibility, and the influence of language/runtime and RTOS scheduling choices. The closest studies nevertheless address these dimensions separately: compiled-versus-interpreted execution benchmarks do not instrument a complete sensing path; RTOS gateways emphasize deterministic task isolation; resource-scalability studies focus on memory or command success; and broker/link studies largely report aggregate communication metrics. The distinguishing feature of the present study is therefore narrower and diagnostic rather than universal: packet-level instrumentation is used to separate node-to-gateway delivery, gateway-side processing, and backend-ingestion timing in a cooperative ESP32–MicroPython gateway, with independent hardware validation constraining the interpretation of the local metric. The quantitative campaign then compares this same bridge-assisted implementation under controlled one-, three-, and five-node periodic workloads. This positioning deliberately avoids claiming that MicroPython outperforms C/C++ or FreeRTOS; instead, it characterizes the timing behavior and limitations of a lightweight interpreted gateway architecture that is underrepresented in existing measurements.

## 2. Materials and Methods

### 2.1. System Architecture

The proposed system is based on a distributed edge–cloud architecture designed for near-real-time environmental monitoring under constrained communication conditions. The architecture consists of multiple autonomous sensor nodes, a central ESP32 gateway, a local MQTT bridge host, a cloud MQTT broker, a Raspberry Pi-based Telegraf service, and an InfluxDB data storage pipeline. Each sensor node performs data acquisition and preliminary processing at the edge and sends telemetry frames to the gateway using a lightweight wireless mechanism operating in the 2.4 GHz ISM band. This approach enables direct device-to-gateway communication while minimizing local protocol overhead and eliminating the need for each sensor node to join the Wi-Fi infrastructure.

**Figure 1:**
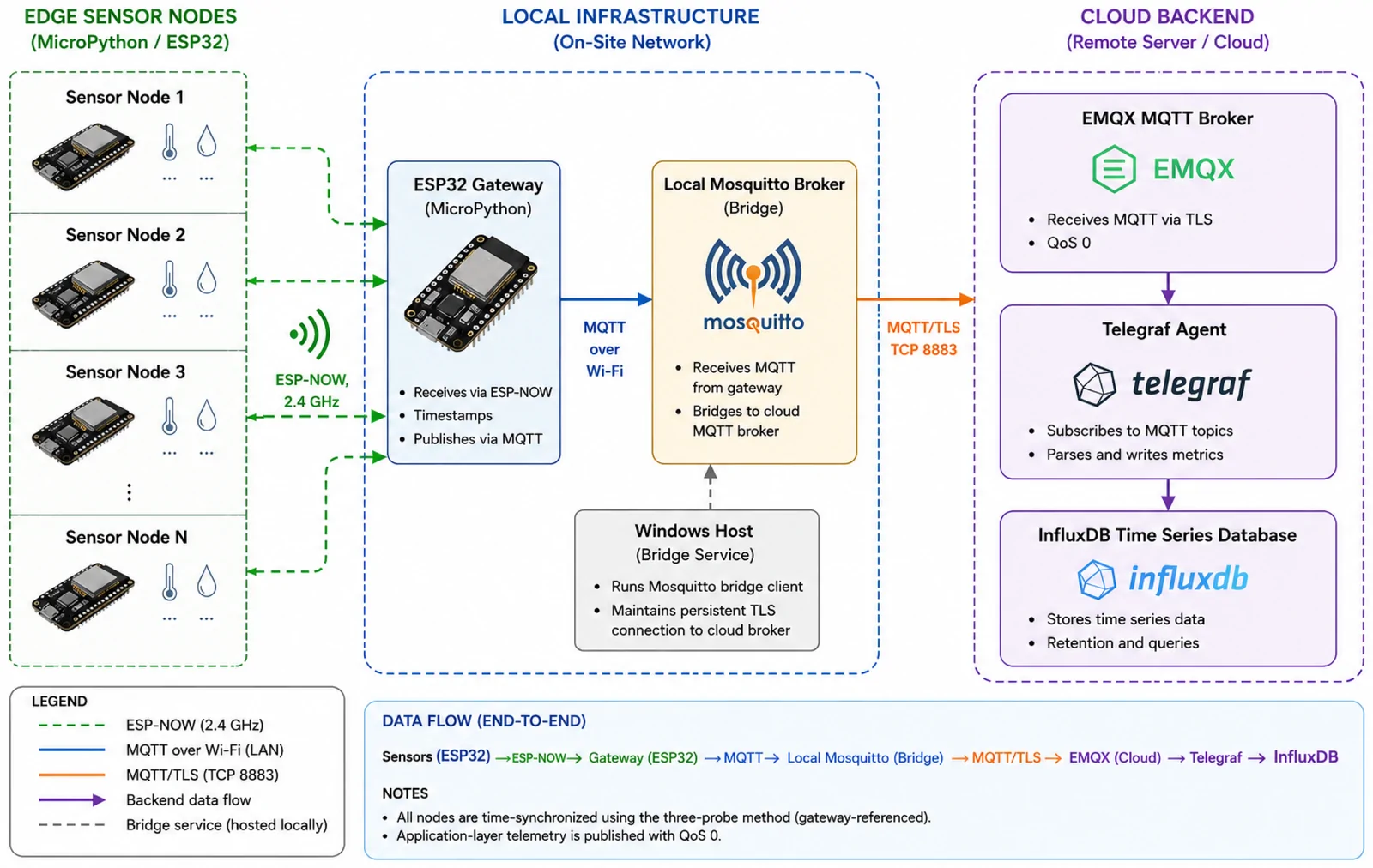
Bridge-assisted edge–cloud IoT system architecture used in the evaluated measurement configuration. Sensor nodes communicate with the ESP32 gateway via ESP-NOW over the 2.4 GHz link. The gateway publishes plaintext MQTT over Wi-Fi/LAN to the local Mosquitto broker on the Windows host; the broker bridges the stream to EMQX Cloud over MQTT/TLS, after which Telegraf on the Raspberry Pi consumes the cloud topic and writes the records to InfluxDB Cloud Serverless.

The ESP32 gateway serves as the local aggregation and timestamping point. It receives telemetry from all active nodes, appends gateway-side reception and publication timestamps, and forwards the packets using MQTT. In the initial direct-cloud configuration, the gateway attempted to publish directly to the cloud MQTT broker using TLS. During development, this direct TLS path occasionally blocked the MicroPython event loop under multi-node load. Therefore, the measurements reported here use a bridge-assisted architecture: the ESP32 gateway publishes plaintext MQTT over the controlled laboratory LAN to a local Mosquitto v2.1.2 broker running on a Windows host, and this host forwards the same topic stream to the EMQX Cloud v5 broker using TLS. A Telegraf v1.36.0 service running on a Raspberry Pi 5 (Raspberry Pi Ltd., Cambridge, UK) subscribes to the EMQX topic and forwards parsed JSON records to InfluxDB Cloud Serverless (Storage Engine Version 3). This arrangement preserves the EMQX–Telegraf–InfluxDB ingestion path while moving TLS termination and cloud forwarding away from the resource-constrained MicroPython gateway. The evaluated bridge-assisted system architecture is shown in Figure 1.

### 2.2. Hardware Platform

The experimental setup consists of multiple custom-designed sensor nodes, a central ESP32 gateway unit, and a backend support host. The sensor nodes and gateway use the Espressif ESP-WROOM-32 module (ESP32-D0WDQ6 SoC; Espressif Systems, Shanghai, China). Each sensor node is built on a microcontroller-based platform and is equipped with sensors for measuring temperature, humidity, and ambient light intensity.

The nodes were implemented as dedicated printed circuit boards (PCBs), designed to support low-power operation and efficient wireless communication. In the current study, five sensor nodes were deployed, forming a small-scale distributed sensing network representative of a controlled experimental testbed.

The gateway is implemented using an ESP32-based node with Wi-Fi connectivity. It acts as an interface between the node-to-gateway communication layer and the backend infrastructure, handling data aggregation, two-way timestamp synchronization, queue management, and MQTT forwarding. In the bridge-assisted campaigns, a Windows host running Mosquitto v2.1.2 was used as the local bridge between the ESP32 gateway and the EMQX Cloud v5 broker. Telegraf v1.36.0 was not executed on this Windows host; it was run as a separate service on a Raspberry Pi, where it consumed the cloud MQTT stream and wrote the parsed records to InfluxDB Cloud Serverless (Storage Engine Version 3).

All firmware for both sensor nodes and the gateway is written in MicroPython v1.24.0 [21,37], a lean implementation of Python 3 targeting microcontrollers. MicroPython was selected due to its support for rapid prototyping, cooperative multitasking via the uasyncio library, JSON handling via ujson, and the availability of a non-blocking driver for the local ESP-NOW-based 2.4 GHz wireless interface enabling asynchronous packet reception within the same event loop. The entire gateway logic, including wireless packet reception, queue management, timestamping, MQTT publication, and NTP-based time synchronization, is implemented as a set of coroutines scheduled by a single uasyncio event loop, with no native threads or RTOS tasks involved. The repeated bridge-assisted multi-node results reported in this version deliberately avoid TLS processing inside this event loop by using the local bridge.

**Figure 2:**
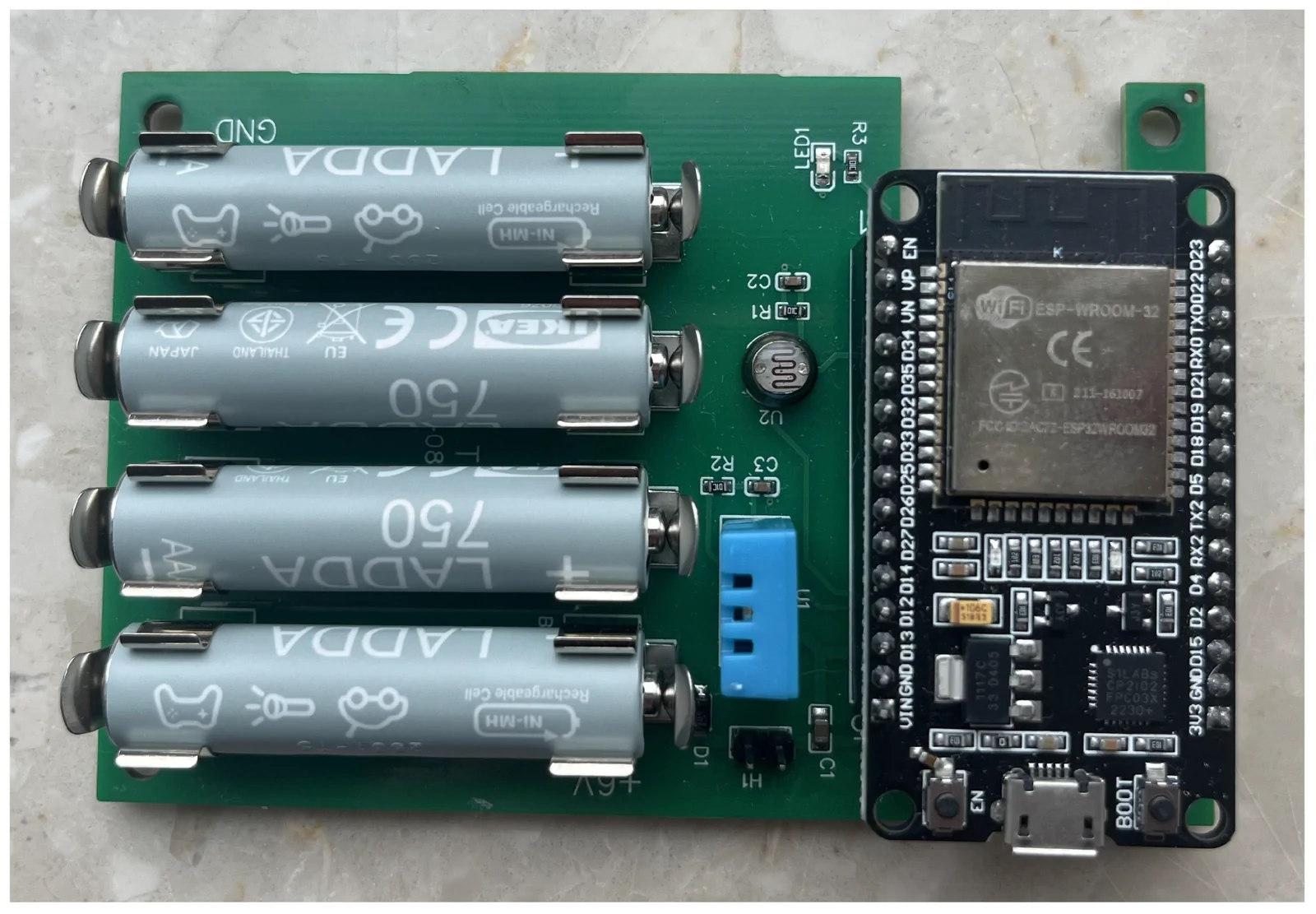
Prototype of the developed sensor node, including the Espressif ESP-WROOM-32 module (ESP32-D0WDQ6 SoC), environmental sensors, and battery-powered supply system implemented on a custom-designed PCB.

A photograph of the developed sensor node hardware platform is shown in Figure 2.

### 2.3. Communication and Data Pipeline

Communication between sensor nodes and the gateway is performed using ESP-NOW, a lightweight connectionless communication mechanism operating in the 2.4 GHz ISM band and available on ESP32-class devices. ESP-NOW was selected because previous comparative studies report favorable latency, range, and energy properties for short local IoT links when compared with conventional Wi-Fi or Bluetooth configurations [19,20]. This solution enables efficient and low-overhead data exchange by avoiding connection-oriented communication models in the local node-to-gateway segment.

The evaluated bridge-assisted configuration forwards collected data using MQTT: the ESP32 gateway publishes plaintext MQTT over the controlled laboratory LAN to a local Mosquitto v2.1.2 broker, and the host forwards the same topic stream to EMQX Cloud v5 over TLS. MQTT publication from the gateway uses QoS 0. The motivation for using this forwarding configuration is summarized in Section 2.5.

The data stream is processed using Telegraf v1.36.0 [38], running on a Raspberry Pi 5 (Raspberry Pi Ltd., Cambridge, UK), which parses incoming MQTT JSON messages from the EMQX Cloud v5 topic and forwards them to InfluxDB Cloud Serverless (Storage Engine Version 3) [39]. For the latency-focused experiments reported here, the backend uses the LATENCY profile with a flush interval of 1 s and flush jitter of 0 s.

To support packet-level performance analysis, telemetry payloads were extended with experimental metadata. In addition to environmental measurements, each packet includes a node identifier (node_id), a per-node sequence number (seq), a unique packet identifier (packet_id), a transmission-mode label (mode), and sender-side timing fields. At the gateway, packet reception time (t_gw_rx_unix_ms) and MQTT forwarding time (t_gw_tx_unix_ms) are appended before publication. In bridge-assisted runs, t_gw_tx_unix_ms denotes the moment immediately before the ESP32 publishes to the local Mosquitto broker; therefore, the backend component includes local broker delivery, bridge forwarding to EMQX, delivery to the Raspberry Pi Telegraf consumer, Telegraf handling, and InfluxDB ingestion. This instrumentation allows latency to be decomposed into node-to-gateway, gateway, and backend segments using only the data stored in InfluxDB, without requiring any additional out-of-band logging.

### 2.4. Time Synchronization and Latency Estimation

To estimate packet-level timing across devices without requiring each sensor node to maintain an independent Internet-synchronized clock, the system uses gateway-referenced synchronization. The gateway synchronizes to NTP before each latency-focused run and establishes a Unix-time anchor associated with its local monotonic timer. Periodic NTP updates are disabled during an individual measurement run to avoid discontinuities caused by clock steps. Sensor nodes retain their own local monotonic timers and estimate the gateway-minus-node clock offset through a lightweight two-way ESP-NOW exchange.

The revised synchronization procedure is performed in exclusive three-probe cycles. During the synchronization probes, new sensor acquisition and telemetry transmission are paused. If a blocking sensor acquisition is already in progress when synchronization becomes due, the synchronization task waits for the acquisition to complete; the completed measurement is retained locally and is timestamped and transmitted only after a fresh synchronization cycle has succeeded. This prevents the synchronous sensor read from extending the request–response timing exchange.

For probe *j*, the node records the local send timestamp $t_{1,j}$ and transmits a synchronization request containing this value and a request sequence identifier. Immediately after receiving the request, the gateway records $t_{2,j}$ using the same gateway monotonic-to-Unix anchor that is later used for telemetry reception timestamps. Immediately before constructing the synchronization response, the gateway records $t_{3,j}$. When the matching response is received by the node, the node records $t_{4,j}$. The application-level round-trip delay excluding gateway response construction time is

(1) $$d_{j}=\left(t_{4,j}-t_{1,j}\right)-\left(t_{3,j}-t_{2,j}\right).$$

Probes for which the gateway is not NTP-synchronized, for which $d_{j}<0$, or for which $d_{j}$ exceeds 100 ms are rejected. Three probes are attempted in each synchronization cycle, with a 120 ms inter-probe gap. A response timeout of 700 ms is used for each probe. Invalid or timed-out probes are discarded. The interval intersection is formed from all valid probes obtained in the current cycle; therefore, a cycle may remain usable when fewer than three responses are valid. A cycle is accepted when at least one valid probe is available and the resulting intersection is non-empty. If no usable intersection is obtained, telemetry remains paused and the complete probe cycle is retried after 1 s.

A conventional NTP-style midpoint estimate,

(2) $${\hat{\theta }}_{j}=\frac{\left(t_{2,j}-t_{1,j}\right)+\left(t_{3,j}-t_{4,j}\right)}{2},$$

is retained as a diagnostic quantity but is not used directly as the active clock correction. The midpoint estimate implicitly assumes sufficiently similar forward and reverse path delays and can therefore be biased by application-level path asymmetry. Instead, each valid probe is treated as defining an admissible interval for the true gateway-minus-node clock offset θ:

(3) $$t_{3,j}-t_{4,j}\leq \theta \leq t_{2,j}-t_{1,j}.$$

For the valid probes in the current synchronization cycle, the node computes the intersection

(4) $$I_{\theta }=\left[\max_{j}\left(t_{3,j}-t_{4,j}\right),\min_{j}\left(t_{2,j}-t_{1,j}\right)\right].$$

A cycle is accepted only when this intersection is non-empty. The active gateway-referenced offset is selected as the midpoint of the common interval,

(5) $$\theta_{\mathrm{sel}}=\frac{\theta_{\mathrm{low}}+\theta_{\mathrm{high}}}{2},$$

where $\theta_{\mathrm{low}}$ and $\theta_{\mathrm{high}}$ are the lower and upper bounds of $I_{\theta }$, respectively. The interval width,

(6) $$w_{\theta }=\theta_{\mathrm{high}}-\theta_{\mathrm{low}},$$

is stored as a synchronization-uncertainty diagnostic. This procedure does not claim hardware-level clock synchronization; rather, it avoids treating path symmetry as an exact assumption and exposes the remaining ambiguity of the application-level offset estimate.

The synchronization cycle is repeated approximately every 20 s. In multi-node experiments, the start of the synchronization process is deterministically de-phased using the node identifier so that several nodes are less likely to initiate three-probe exchanges simultaneously. The synchronization policy is otherwise identical for the one-, three-, and five-node scenarios.

After a successful synchronization cycle, a telemetry sample is timestamped only after sensor acquisition has completed. The node first captures its local monotonic timestamp $t_{\mathrm{node},\mathrm{ticks}}$ and then forms the gateway-referenced sender timestamp

(7) $$t_{\mathrm{node}}=t_{\mathrm{node},\mathrm{ticks}}+\theta_{\mathrm{sel}}.$$

**Figure 3:**
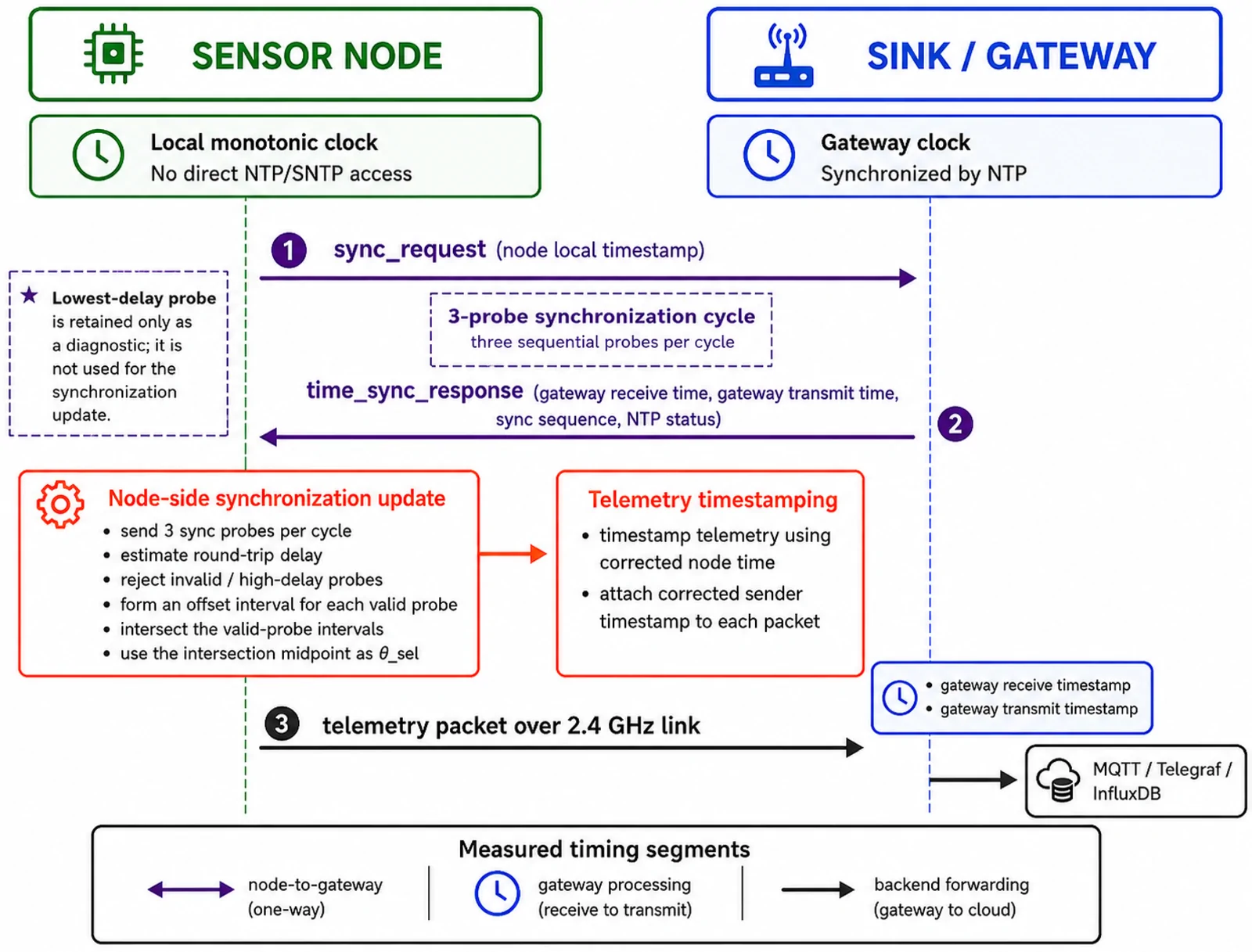
Three-probe gateway-referenced synchronization and packet-level timestamping procedure. Each synchronization cycle attempts three sequential two-way probes. The admissible clock-offset intervals from the valid probes are intersected and the midpoint of the non-empty intersection is used as $\theta_{\mathrm{sel}}$; the lowest-delay probe is retained only as a diagnostic. Telemetry is timestamped and transmitted after successful synchronization.

The compact telemetry payload is constructed only after this timestamp has been captured. The gateway records the local reception timestamp immediately after await e.arecv() returns, before JSON parsing or queue processing, and later records the MQTT publication timestamp. The backend timestamp is the ingestion timestamp assigned by the Telegraf MQTT-consumer path. Four timestamps are therefore available for each valid telemetry packet: $t_{\mathrm{node}}$, $t_{\mathrm{gw},\mathrm{rx}}$, $t_{\mathrm{gw},\mathrm{tx}}$, and $t_{\mathrm{cloud}}$. The synchronization and packet-level timestamping procedure is summarized in Figure 3.

Based on these timestamps, the following latency components are defined:

- Node-to-gateway delivery latency:
  (8) $$L_{n2g}=t_{\mathrm{gw},\mathrm{rx}}-t_{\mathrm{node}}$$
- Gateway processing latency:
  (9) $$L_{\mathrm{gateway}}=t_{\mathrm{gw},\mathrm{tx}}-t_{\mathrm{gw},\mathrm{rx}}$$
- Gateway-to-backend ingestion latency:
  (10) $$L_{\mathrm{backend}}=t_{\mathrm{cloud}}-t_{\mathrm{gw},\mathrm{tx}}$$
- End-to-end ingestion latency:
  (11) $$L_{\mathrm{total}}=t_{\mathrm{cloud}}-t_{\mathrm{node}}.$$

For readability, Table 2 summarizes the packet-level metrics used in the revised campaign.

The backend timestamp $t_{\mathrm{cloud}}$ is assigned when the MQTT consumer creates the metric for the ingestion pipeline; it is not a hardware timestamp and is not interpreted as the physical database-commit time. No fixed compensation constant is subtracted from any latency component.

#### Independent Oscilloscope Validation of the Node-to-Gateway Timing Metric

An independent hardware timing experiment is used to quantify the agreement between the software-derived $L_{n2g}$ metric and a clock-independent reference. The validation is performed with one sensor node and the same sender and gateway firmware used for the revised campaign, with GPIO timing markers enabled only for the validation runs.

On the sender, GPIO18 is driven high immediately after $t_{\mathrm{node},\mathrm{ticks}}$ and the corresponding gateway-referenced $t_{\mathrm{node}}$ have been captured and before the telemetry payload is constructed. On the gateway, GPIO33 is driven high immediately after the ESP-NOW receive operation returns and the gateway reception timestamp $t_{\mathrm{gw},\mathrm{rx}}$ is captured. The two signals are observed simultaneously with a RIGOL DHO1204 200 MHz oscilloscope (RIGOL Technologies Co., Ltd., Suzhou, China). The primary hardware quantity is the rising-edge-to-rising-edge delay from the sender marker to the corresponding gateway marker.

The gateway marker is generated for received ESP-NOW frames. Synchronization exchanges do not create an ambiguity in the telemetry measurement because sensor acquisition and telemetry transmission are paused while the three-probe synchronization cycle is active. Thus, a sender telemetry marker can be paired with the first subsequent gateway receive marker for that telemetry event.

Three independent 15 min validation runs were performed. The sender and gateway are restarted between runs so that each run includes a new initialization and synchronization sequence. Firmware, device placement, radio channel, periodic traffic policy, and oscilloscope settings are kept unchanged. For each run, the oscilloscope-reported mean rising-edge delay is compared with the mean software-derived $L_{n2g}$ computed from the InfluxDB records acquired over the same time window. Because the oscilloscope data are used here as aggregate run-level statistics rather than packet-paired exports, the primary agreement measure is the difference between run means,

(12) $$\Delta_{\mathrm{SW}-\mathrm{HW}}={\bar{L}}_{n2g,\mathrm{SW}}-{\bar{L}}_{n2g,\mathrm{HW}},$$

rather than a packet-level mean absolute error. The software median and p95, the number of analyzed telemetry packets, and the median synchronization interval width $w_{\theta }$ are also retained to characterize the software distribution and the residual synchronization ambiguity. The oscilloscope measurements are used only for independent validation; they are not used to calibrate the wireless synchronization or to derive a correction constant for $L_{n2g}$.

### 2.5. Gateway Forwarding Configuration

During system development, direct MQTT/TLS forwarding from the MicroPython gateway was found to be insufficiently robust for a reproducible multi-node campaign. The quantitative experiments reported in this work therefore use the bridge-assisted configuration shown in Figure 1. In this configuration, the ESP32 gateway publishes plaintext MQTT over the controlled laboratory LAN to a local Mosquitto broker, while TLS-secured forwarding to EMQX Cloud is performed by the host. Direct-cloud operation is retained only as robustness context and is not used as a quantitative latency baseline.

The evaluated gateway firmware uses a publish queue capacity of 500 packets and processes up to four queued packets per scheduling cycle. A cooperative yield is inserted between consecutive publications and the idle sleep is 5 ms. Synchronous per-packet CSV logging is disabled during the latency-focused runs. Following a publication failure, the MQTT client reference is cleared, the failed packet is retained for retry, and reconnection is attempted without an additional blocking back-off delay. These settings were kept unchanged throughout the bridge-assisted one-, three-, and five-node campaign.

### 2.6. Experimental Setup and Evaluation Metrics

The revised evaluation uses a real hardware testbed consisting of up to five ESP32 sensor nodes and one ESP32 gateway in a controlled indoor environment. The main quantitative campaign evaluates one, three, and five simultaneously active sensor nodes using the bridge-assisted forwarding path. Three independent 30 min repetitions were completed for each node-count scenario. The local ESP-NOW communication mechanism, compact payload schema, three-probe gateway-referenced synchronization procedure, periodic sender mode, queue capacity, publish-batch size, sender jitter policy, MQTT QoS, and backend LATENCY profile were kept unchanged across node counts. The physical positions and radio geometry of the gateway and sensor nodes were also kept unchanged. The ESP-NOW channel was configured to match the Wi-Fi channel used by the gateway in the corresponding run; channel matching was therefore a connectivity setting rather than an intentionally varied experimental factor.

Table 3 lists the main implementation and campaign parameters for the revised bridge-assisted experiment.

The nominal 1 s value is an inter-cycle sleep rather than a fixed 1 Hz transmission period. In the measured implementation, DHT acquisition required approximately 273 ms on average, while synchronization pauses, payload processing, and ESP-NOW transmission add further overhead. Consequently, the observed sender rate was approximately 0.77 packets/s per node. This measured rate, rather than an assumed 1 Hz rate, is used for throughput analysis.

The LATENCY backend profile is retained for all main latency comparisons. The Telegraf MQTT consumer operates as a service input and receives MQTT messages as they arrive; the generic polling interval therefore does not define MQTT-consumer sampling. The backend timestamp used here is the timestamp assigned by the ingestion pipeline, while the output flush configuration affects subsequent write timing and visibility.

The primary database-visible packet delivery ratio is computed using the exact sender-side number of ESP-NOW telemetry transmission attempts reported in the final run summary:

(13) $$PDR_{\mathrm{stored}}=\frac{N_{\mathrm{unique},\mathrm{Influx}}}{N_{\mathrm{TX},\mathrm{attempt}}}\times 100\%.$$

The final run summary is transmitted repeatedly after the measured window to improve the probability that the denominator is captured. Per-node sequence continuity, missing sequence values, duplicate/reordered records, gateway receive counts, gateway publish counts, and queue-drop counters are reported as independent consistency checks. If an exact sender run summary is unavailable, that run is not used for the exact-denominator PDR summary, although its sequence-continuity diagnostics may still be inspected.

Throughput is evaluated at four successive telemetry stages: sender transmission (TX), gateway reception (RX), gateway MQTT publication, and database storage. For stage *k* in run *r*, aggregate throughput is defined as

(14) $$R_{k,r}=\frac{N_{k,r}}{T_{r}},$$

where $N_{k,r}$ is the number of telemetry packets observed at stage *k* and $T_{r}$ is the measured run duration in seconds. The common aggregate run duration is taken as the longest final sender runtime among the active nodes, which is approximately 30 min in every run. Per-node throughput is calculated analogously,

(15) $$R_{k,r,j}=\frac{N_{k,r,j}}{T_{r,j}},$$

using the final runtime $T_{r,j}$ reported by node *j*. Database-stored counts are deduplicated by the node_id–seq pair before throughput calculation.

The final gateway RX and publication counters include a small number of post-run summary/control frames in addition to telemetry. They are therefore retained as audit counters rather than interpreted as independent telemetry-stage denominators. Sender ACK equality, zero sender send errors, zero gateway queue drops, and zero recorded MQTT publication failures are reported as consistency diagnostics, but a MAC-layer ESP-NOW acknowledgement is not treated as proof that the gateway application processed every telemetry packet. The primary independently defined throughput comparison is therefore sender offered rate versus database-stored rate. The complete five-node RUN02 and RUN03 sequence ranges were reconstructed from the full-day InfluxDB export using each node’s final run summary and nearest preceding sequence reset (seq = 1), so exact full-run database-stored throughput and PDR are available for all five-node repetitions.

Scenario-level latency summaries are based on independent runs rather than treating all packet observations as independent experimental replicates. Packet-level distributions are retained for descriptive empirical cumulative distribution function (ECDF) and percentile analysis, while run-level summaries are used for between-condition interpretation. For a given node-count scenario *s*, the descriptive packet-level ECDF is computed from all valid $L_{n2g}$ observations pooled across its three independent 30 min repetitions:

(16) $${\hat{F}}_{s}\left(x\right)=\frac{1}{N_{s}}\sum_{i=1}^{N_{s}}1\;\left(L_{n2g,i}\leq x\right),$$

where $N_{s}$ is the number of valid packet observations in scenario *s* and 1(·) is the indicator function. Thus, ${\hat{F}}_{s}\left(x\right)$ gives the observed fraction of packets whose gateway-referenced local delivery latency does not exceed *x*. The pooled ECDFs are used only to visualize the packet-level distribution shape; the individual packets are not treated as independent experimental replicates for statistical inference.

Before ECDF construction, the same inclusion rules used for the latency tables are applied: records must contain a valid $L_{n2g}$ value and belong to the configured node set of the measured run, and duplicate node_id–seq records are removed by retaining the first stored instance. When a broad InfluxDB export contains a short tail from a neighboring acquisition window, only the node IDs belonging to the configured run are retained. No latency observations are removed solely because they are large; transient tail events therefore remain represented in the ECDF. This avoids pseudo-replication while preserving the distributional information requested by the reviewers.

For between-scenario comparisons, one 30 min run is treated as one independent experimental replicate. For each run *r*, packet-level observations are first reduced to run-level descriptors, including the mean, median, and p95 of $L_{n2g}$. The scenario value is then summarized from the three independent run-level values as mean ± SD. To quantify the magnitude of the node-count effect without inflating the sample size by treating packets as independent replicates, the primary effect sizes are reported as the absolute difference between scenario-level run means,

(17) $$\Delta_{A\to B}={\bar{M}}_{B}-{\bar{M}}_{A},$$

and the corresponding relative change,

(18) $$\Delta_{\%,A\to B}=100\frac{{\bar{M}}_{B}-{\bar{M}}_{A}}{{\bar{M}}_{A}},$$

where *M* denotes the selected run-level latency descriptor. A 95% Student-*t* confidence interval is additionally computed for the mean of the three run-level values in each scenario. Because only n=3 independent repetitions are available per condition, these intervals and standardized effect-size estimators are considered descriptive rather than precise population-level estimates. Accordingly, the Results emphasize raw differences in milliseconds, percentage changes, and the consistency of the three independent repetitions.

### 2.7. Experimental Run Protocol and Data Inclusion Criteria

All revised campaign scenarios follow a fixed run protocol. Before each measured run, the system is initialized and observed for a warm-up interval of 2 min to 5 min; warm-up samples are excluded from statistical reporting. The measured duration is 30 min per run for the one-node, three-node, and five-node scenarios.

Sender nodes operate in periodic mode with a nominal 1 s interval. Telemetry does not begin until the first successful three-probe synchronization cycle has completed. During later synchronization cycles, new sensing and telemetry transmission are paused until a valid current-cycle offset interval has been obtained. Sensor acquisition itself is intentionally outside the definition of $L_{n2g}$: $t_{\mathrm{node}}$ is captured only after the sensor read and after any required synchronization pause.

For all node counts, each sender uses the same source-side de-synchronization policy: a deterministic startup offset derived from node identity and the same bounded per-interval jitter. The jitter policy is not changed specifically for the five-node scenario. Synchronization-cycle start times are additionally deterministically de-phased between nodes to reduce simultaneous three-probe bursts.

Samples are included in latency statistics only when the required timestamps and run metadata are present and parseable. Duplicate records are identified using the node_id–seq pair; the first stored instance is retained for latency statistics and duplicate counts are reported separately. Runs with persistent backend authorization failures, missing exact run denominators, incomplete synchronization diagnostics, or known gateway failures are flagged before comparative analysis.

The main bridge-assisted campaign used a frozen firmware revision once validation was complete. Oscilloscope marker instrumentation was enabled only for the dedicated validation runs described in Section Independent Oscilloscope Validation of the Node-to-Gateway Timing Metric; the normal one-, three-, and five-node campaign runs were performed without the oscilloscope marker tasks.

## 3. Results

The revised results below use only the final three-probe synchronization/measurement procedure. Numerical values obtained with the earlier synchronization implementation are not carried forward into the main latency analysis.

### 3.1. Data Continuity and Database-Visible Packet Delivery

The final instrumentation uses sender-side transmission-attempt counters as the primary reliability denominator, with gateway counters and database sequence continuity used as consistency checks. After reconstruction of the complete five-node sequence windows from the full-day InfluxDB export and removal of duplicate node–sequence records by retaining the first stored instance, exact full-run database-visible PDR is available for every bridge-assisted run. All three single-node runs achieved 100% PDR. The three-node runs achieved 99.281%, 99.880%, and 99.397%, whereas the five-node runs achieved 96.900%, 96.747%, and 97.047%, respectively. Sender run summaries reported 100% ESP-NOW acknowledgements, zero negative acknowledgements, and zero send errors in the included runs. Gateway diagnostics reported no RX/TX queue drops and no MQTT publication failures. The stage-specific reliability results are summarized in Table 4.

The gateway diagnostics showed no sustained queue accumulation or recorded gateway-side queue loss. One five-node run contained four unusually large $L_{n2g}$ observations temporally close to a synchronous MQTT publication whose console-recorded duration reached approximately 1.59 s; these observations are retained. This temporal association is discussed as a plausible cooperative-scheduling mechanism rather than treated as proof of causation.

### 3.2. Aggregate and Per-Node Throughput

The offered telemetry rate increased approximately in proportion to the number of active nodes. Mean sender TX throughput across the three independent repetitions was 0.7670 ± 0.0003 packets/s for one node, 2.3093 ± 0.0062 packets/s for three nodes, and 3.8547 ± 0.0034 packets/s for five nodes. The corresponding exact database-visible stored rates were 0.7670 ± 0.0003, 2.2982 ± 0.0083, and 3.7351 ± 0.0056 packets/s. Run-level throughput values are reported in Table 5.

**Figure 4:**
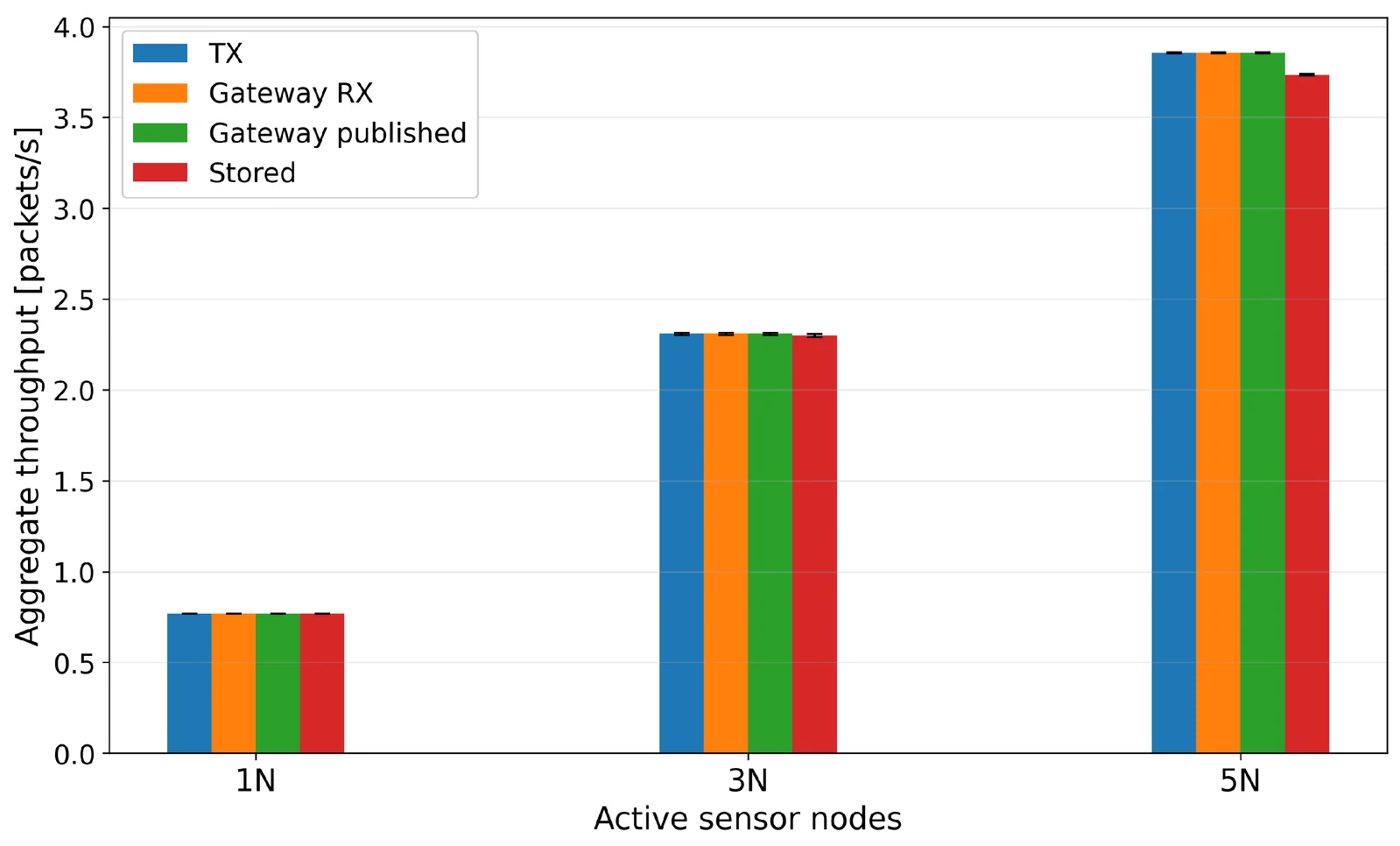
Aggregate sender offered rate and database-stored telemetry rate for the one-, three-, and five-node bridge-assisted scenarios. Bars show means across three independent 30 min runs.

The per-node offered rate remained close to 0.77 packets/s across node counts, confirming that aggregate load increased primarily by adding comparable node streams rather than by changing the cadence of individual senders. The lower stored rate at five nodes is therefore a database-visible delivery effect under the evaluated end-to-end pipeline rather than an intentionally reduced offered load. Successful sender ESP-NOW acknowledgement and the absence of recorded gateway queue/publication errors do not identify the precise downstream loss location, particularly because gateway MQTT publication used QoS 0; they only show that the missing database records were not accompanied by the recorded local error conditions. The aggregate sender and database-stored rates, together with gateway audit counters, are shown in Figure 4.

### 3.3. Latency Decomposition

The run, rather than the individual packet, is treated as the independent experimental replicate. Packet-level medians and p95 values are reported descriptively to expose the distribution shape without treating thousands of packets as independent samples.

**Figure 5:**
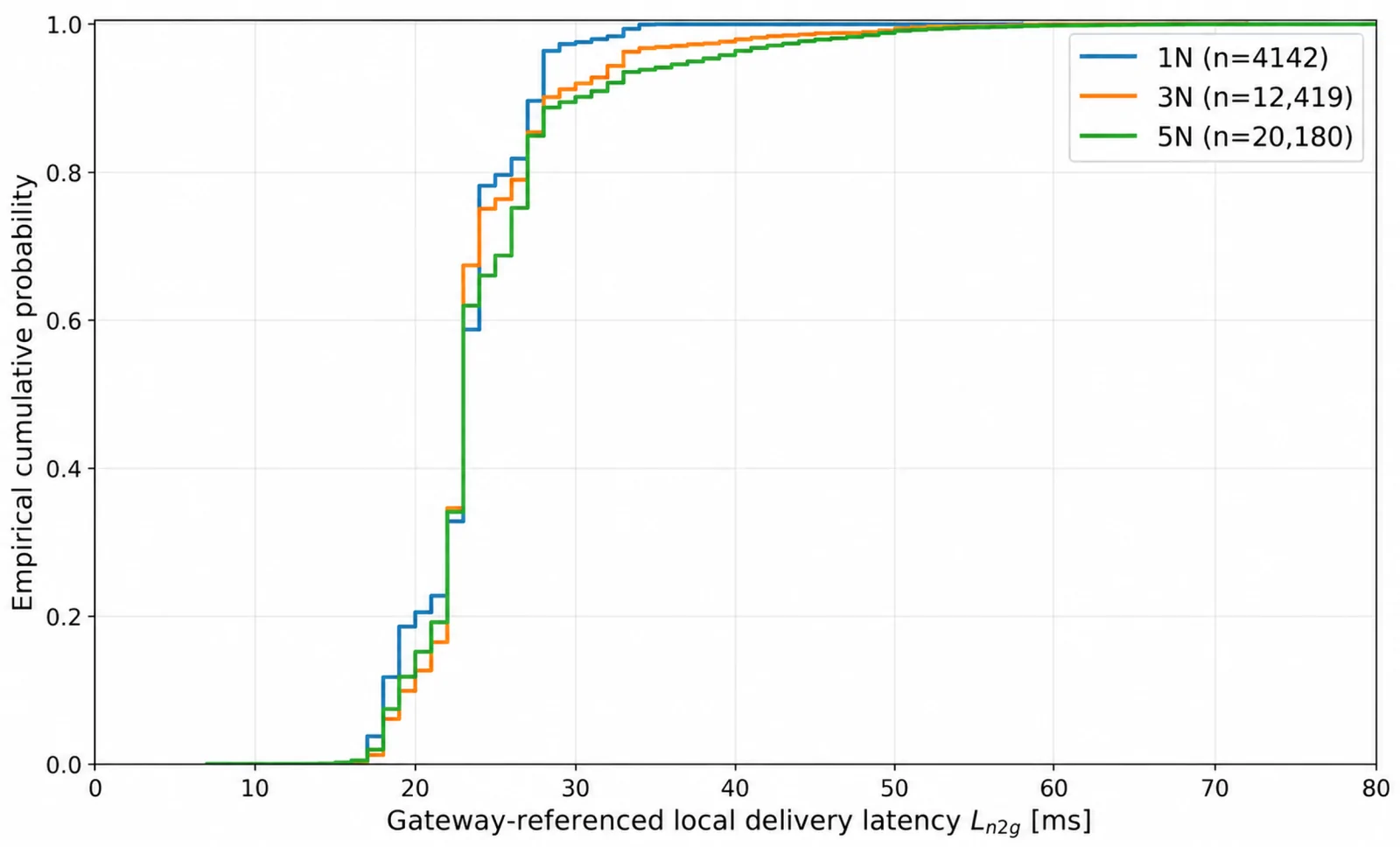
Packet-level empirical cumulative distribution functions (ECDFs) of the gateway-referenced local delivery latency $L_{n2g}$ for the bridge-assisted one-, three-, and five-node scenarios. For each scenario, valid packet observations from the three independent 30 min repetitions are pooled only for descriptive distribution visualization. The corresponding sample sizes after duplicate removal are *N* = 4142, 12,419, and 20,180 packets for 1, 3, and 5 nodes, respectively. The x-axis is limited to 80 ms to resolve the main body of the distributions; all valid observations, including larger tail values, are retained in the ECDF calculation.

Figure 5 complements the run-level summary by showing the full packet-level distribution rather than only selected percentiles. The three curves overlap strongly around the center of the distribution: the pooled median remains 23 ms for all three node-count scenarios. The main change with increasing node count is therefore not a large displacement of the entire distribution, but a progressive broadening of its upper tail. The pooled p95 increases from 28 ms for one node to 33 ms for three nodes and 38 ms for five nodes, while the corresponding p99 values are 33, 49, and 50 ms. Consequently, approximately 95% of one-node packets are delivered locally within 28 ms, whereas the same cumulative fraction is reached at approximately 33 ms and 38 ms for the three- and five-node scenarios.

This distributional view supports the interpretation obtained from the independent run summaries. Adding active nodes causes only a modest change in the typical local delivery time, but increases the probability of packets experiencing longer application-level delays. Because the gateway-processing mean remains nearly unchanged across the same scenarios and the campaign diagnostics do not show sustained queue accumulation, the broader $L_{n2g}$ tail is more consistent with occasional contention, scheduling, or synchronization-related timing variability than with persistent gateway saturation. Rare large observations are retained in the analysis rather than removed as outliers; therefore, the ECDF reflects both the stable central operating regime and the measured tail behavior. The ECDF is descriptive at packet level, whereas the three independent runs per condition remain the experimental replication unit for statistical comparisons.

**Figure 6:**
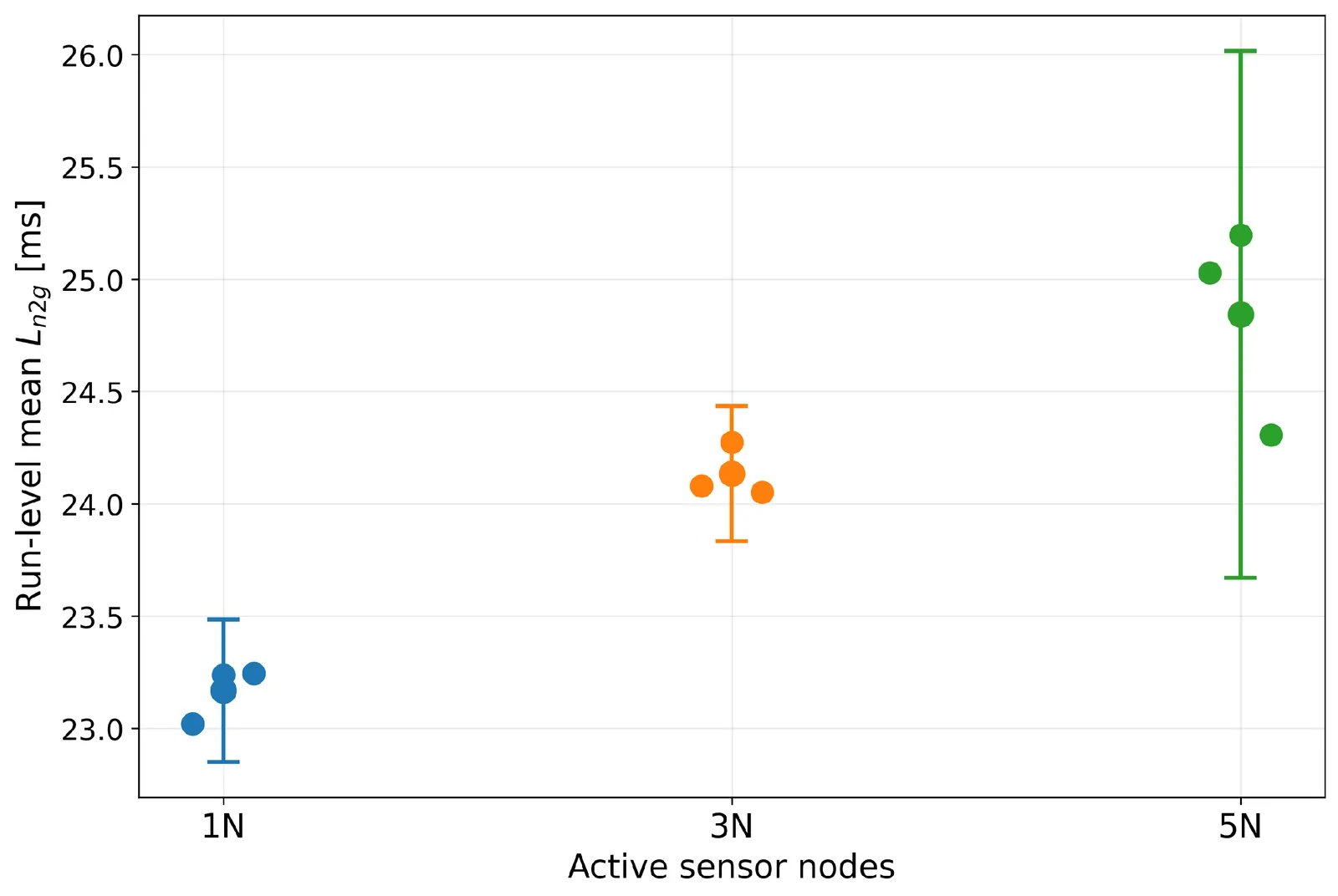
Run-level mean gateway-referenced local delivery latency $L_{n2g}$ for the one-, three-, and five-node bridge-assisted scenarios. Each small point represents one independent 30 min run (n=3 per condition). The larger marker shows the mean of the three run-level means, and the error bar shows the corresponding 95% Student-*t* confidence interval. Packet observations are used only to calculate the mean within a run; they are not treated as independent replicates in this comparison.

Figure 6 makes the experimental replication explicit. The run-level points show a narrow spread for the one- and three-node conditions and somewhat greater between-run variability at five nodes. Scenario-level numerical summaries are reported in Table 6, while the individual run values are retained in Appendix A.

Synchronization-uncertainty diagnostics for the individual runs are summarized in Table 7.

Run-level effect sizes are summarized in Table 8.

The raw run-level effect sizes show that the node-count effect is substantially stronger in the upper latency tail than in the average latency level. Relative to the one-node condition, the five-node condition increased the average of the independent run means by 1.68 ms (7.2%), whereas the average run-level p95 increased by 9.67 ms (34.5%). The run-level median remained 23 ms in every repetition of every scenario, so the observed change is not a uniform shift of the complete latency distribution. Instead, the combined ECDF and run-level analyses consistently indicate a stable central latency accompanied by more frequent higher-delay observations as the number of simultaneously active nodes increases.

The 95% confidence intervals in Figure 6 are intentionally shown as uncertainty summaries over only three independent runs, not as evidence of high-precision population estimates. Likewise, standardized effect sizes can become numerically large when between-run variance is very small at such a small *n*. For this reason, the interpretation is based primarily on the directly observable run-level differences and percentage changes rather than on packet-count-driven significance testing. This treatment preserves the independence structure of the experiment and directly addresses the risk of pseudo-replication.

Taken together, the run-level and distributional results indicate a stable central local-delivery latency with a broader upper tail as node count increases. Gateway-processing latency remains nearly unchanged and no sustained queue growth is observed, whereas the backend-associated component is substantially more variable between runs and is not monotonic with node count.

### 3.4. Independent Hardware Validation of $L_{n2g}$

The revised timestamping procedure is validated independently with the GPIO/ oscilloscope experiment described in Section Independent Oscilloscope Validation of the Node-to-Gateway Timing Metric. The purpose of this experiment is not to replace wireless synchronization during normal system operation, but to quantify the agreement between the software-derived one-way timing metric and an external clock-independent reference.

Table 9 summarizes the three independent 15 min validation runs. The primary comparison is the run-level difference between the software and oscilloscope means. A positive value of $\Delta_{\mathrm{SW}-\mathrm{HW}}$ indicates that the software estimate is larger than the oscilloscope reference.

Across the three runs, the software-derived mean $L_{n2g}$ remained within a narrow range of 26.167–27.328 ms, whereas the oscilloscope-reported mean ranged from 12.638 to 15.899 ms. The software estimate exceeded the hardware reference in all three runs, with run-level differences of +10.268, +12.228, and +13.901 ms. The mean difference was therefore +12.132 ms, with a between-run standard deviation of 1.819 ms. The sign consistency across independent restarts indicates a reproducible positive bias of the software-derived one-way latency estimate under the evaluated conditions.

The oscilloscope reference is independent of the node/gateway clock-offset estimate, whereas the software value includes residual uncertainty associated with application-level timestamp placement and wireless clock alignment. The median synchronization interval width $w_{\theta }$ was 28–29 ms across the runs, showing that the current application-level synchronization procedure still leaves a non-negligible admissible range for the clock offset. The hardware validation therefore supports use of $L_{n2g}$ as a reproducible gateway-referenced application-level timing metric, but not as an unbiased estimate of the physical one-way radio delivery time. No empirical compensation constant derived from the oscilloscope measurements is applied to the campaign data; the observed bias is instead reported explicitly as a limitation of the software timestamping method.

### 3.5. Direct-Cloud Robustness Observations and Scope of Comparison

Three independent 30 min single-node direct-cloud runs were completed successfully, but stable three-node application-level operation could not be established reproducibly. Representative failures involved synchronization-response timeouts despite successful ESP-NOW transmission acknowledgements, indicating disruption above the local MAC acknowledgement stage without identifying a unique internal cause. The direct diagnostic used TLS without server-certificate verification because strict validation was not operational on the evaluated MicroPython/mbedTLS build. Consequently, these observations are reported only as robustness context; no quantitative direct-cloud versus bridge-assisted latency comparison is made.

## 4. Discussion

The repeated bridge-assisted campaign supports three main interpretations. First, the central local-delivery metric changes modestly with node count while its upper tail broadens. The run-level mean $L_{n2g}$ values were 23.17 ± 0.13 ms, 24.13 ± 0.12 ms, and 24.84 ± 0.47 ms for one, three, and five nodes, respectively, whereas packet-level p95 increased from 28 ms to 33 ms and then 37–38 ms. The median remained 23 ms in every run. This pattern is more consistent with intermittent scheduling/contention effects than with a uniform increase in per-packet service time.

Second, the synchronization diagnostic does not show systematic deterioration with node count. The run-level median $w_{\theta }$ values were 29/29/29 ms for one node, 29/29/29 ms for three nodes, and 23/28/29 ms for five nodes; the corresponding p95 values were 30/30/30, 31/31/30, and 30/30/30 ms. Because $w_{\theta }$ is an admissible interval width rather than a confidence interval, it cannot be directly subtracted from $L_{n2g}$ or used as a formal error bar. Nevertheless, the absence of a systematic increase with node count argues against explaining the broader five-node tail solely by degradation of the offset-interval estimate.

Third, the cooperative MicroPython gateway provides a plausible mechanism for rare local-latency excursions. $L_{\mathrm{gateway}}$ measures the interval from gateway reception to the timestamp immediately before the MQTT publication call; it does not include time spent inside a blocking synchronous client.publish() call. A long publication can therefore prevent the single uasyncio event loop from returning promptly to ESP-NOW reception, causing subsequently arriving packets to receive a late $t_{\mathrm{gw},\mathrm{rx}}$ and making the stall appear in $L_{n2g}$ rather than in $L_{\mathrm{gateway}}$. In one five-node run, four large $L_{n2g}$ observations occurred temporally close to a console-recorded MQTT publication maximum of approximately 1.59 s. This temporal association is consistent with event-loop starvation, but the available instrumentation does not prove a unique causal chain.

The near-constant mean $L_{\mathrm{gateway}}$ (13.31–13.46 ms) and observed queue maxima of only one to two packets indicate that the ordinary receive-to-publication scheduling path did not accumulate a sustained backlog under the tested load. This does not imply that the gateway is behaviorally transparent: synchronous operations can still delay when the next receive coroutine is scheduled, as noted above. The distinction is important because a timestamp boundary can move an implementation-level stall from one nominal latency component into another.

The measured sender rate also explains why a nominal 1 s periodic setting produced approximately 0.77 packets/s per node. The firmware sleeps for approximately 1 s between cycles, but the synchronous sensor acquisition itself requires about 273 ms on average, with synchronization pauses, bounded jitter, payload serialization, and ESP-NOW transmission added outside that sleep. The observed throughput is therefore internally consistent with the sender implementation and should not be interpreted as unexplained loss of offered traffic.

Database-visible reliability exhibits a clearer node-count effect than local acknowledgement. Exact stored PDR remained 100% for one node, 99.28–99.88% for three nodes, and 96.75–97.05% for five nodes. Because sender ACK remained 100% and the gateway recorded zero queue drops and zero MQTT publication failures, the loss is not reflected in those local diagnostics and becomes visible only in the downstream database record stream. QoS 0 prevents the gateway publication call from serving as an end-to-end delivery acknowledgement, so the present data do not justify assigning the missing records to one particular downstream component.

The backend-associated timing component varied much more strongly between runs than the local embedded components. These values are therefore used to demonstrate why stage decomposition is necessary, not as a controlled benchmark of cloud-provider performance or as evidence of node-count scaling. The backend metric spans the local broker/bridge, WAN-facing transport, EMQX, Telegraf ingestion timestamp semantics, and inter-host clock alignment; different network or host conditions can shift it substantially. A future backend-specific campaign should timestamp additional stages on a common or independently validated clock basis.

### Contextual Quantitative Comparison with Related Work

Table 10 places the measured values in the context of selected ESP-NOW, MQTT, and IoT time-synchronization studies. The comparison is not intended as a direct benchmark. The cited works use different hardware, timing definitions, traffic models, and protocol layers. Its purpose is narrower: it shows why the present application-level decomposition should be interpreted separately from pure local-link delay, broker-level MQTT stress tests, and clock-synchronization studies.

The comparison also helps delimit the claim of the manuscript. The reported $L_{n2g}$ values are not intended to replace link-layer ESP-NOW measurements. They are gateway-referenced application-level metrics designed for an end-to-end data-acquisition pipeline. Likewise, the backend numbers are not presented as universal cloud-performance results. They describe the specific bridge-assisted ingestion path used in the experiment and are included so that local gateway behavior is not hidden inside a single aggregate delay value.

## 5. Limitations

The main limitation of the timing method is that synchronization and timestamp capture are implemented in MicroPython software. No hardware timestamping is used during the normal campaign. The $L_{n2g}$ metric therefore includes residual synchronization/path-asymmetry error, timestamp-placement effects, sender-side packet preparation/scheduling, receiver-side scheduling, and local wireless delivery. The independent oscilloscope validation demonstrates a reproducible positive software-to-hardware difference and constrains the metric’s interpretation; it does not justify a universal post hoc correction constant.

The three-probe synchronization procedure intersects the admissible offset intervals from the valid probes in the current cycle and uses the midpoint of the non-empty intersection as the active estimate. The interval width $w_{\theta }$ is an uncertainty diagnostic rather than an empirical confidence interval. Path asymmetry can still bias an individual corrected node timestamp even when the intersection is non-empty.

The experiments were performed in a controlled indoor environment with at most five active nodes, small telemetry payloads, periodic traffic, and MQTT QoS 0. They do not establish general scalability to larger deployments, burst traffic, stronger 2.4 GHz interference, larger payloads, higher MQTT QoS levels, or deliberate broker-disconnection scenarios. Each bridge-assisted scenario was repeated three times, so run-level standard deviations are descriptive measures of between-run variability rather than a basis for strong formal statistical inference.

A compiled C/C++ or FreeRTOS gateway implementation was not developed as a controlled comparator. The present results therefore characterize the evaluated MicroPython implementation and should not be interpreted as evidence that MicroPython is faster or more resource-efficient than compiled alternatives. Direct CPU utilization and energy consumption were also not measured in the final low-overhead campaign.

The backend component is architecture-specific. It includes local broker/bridge handling, WAN-facing TLS transport, EMQX, Telegraf, and InfluxDB ingestion semantics; a different bridge host, operating system, network, cloud region, broker configuration, or ingestion service could change this component substantially. The metric is not a database-write-completion benchmark.

The direct-cloud observations are not a matched multi-node performance comparison. The diagnostic direct runs used TLS encryption without server-certificate verification because strict certificate validation was not operational on the evaluated MicroPython/mbedTLS build. Although three single-node 30 min runs completed, stable three-node application-level operation could not be established reproducibly. The manuscript therefore does not quantify a direct-versus-bridge latency improvement and does not infer a unique internal cause for the direct-cloud failures.

The physical placement and radio geometry were kept fixed, but the ESP-NOW channel was matched to the gateway Wi-Fi channel in each run rather than held to one channel value across the entire campaign. Uncontrolled differences in channel occupancy can therefore contribute to run-to-run wireless variability. The experiments were not designed as a channel-comparison study.

## 6. Conclusions

This work developed and evaluated an ESP32/MicroPython edge–cloud IoT testbed with packet-level timing instrumentation and a three-probe gateway-referenced synchronization procedure. The revised method pauses telemetry during synchronization, intersects admissible clock-offset intervals from the current probe cycle, and exposes the interval width as an uncertainty diagnostic.

The bridge-assisted campaign comprised three 30 min repetitions with one, three, and five active nodes under the same periodic workload. Mean gateway-referenced $L_{n2g}$ increased only modestly across this range (23.17, 24.13, and 24.84 ms across scenario-level run means), whereas the p95 increased from 28 ms to 37–38 ms; mean gateway-processing latency remained nearly unchanged at 13.31–13.46 ms. Within the evaluated workload, the gateway-processing stage therefore showed no evidence of sustained overload, while the upper tail of local delivery latency broadened more visibly than the mean.

Independent GPIO/oscilloscope validation showed a consistent positive software-to-hardware difference, so $L_{n2g}$ is interpreted as a gateway-referenced application-level local delivery metric rather than unbiased physical one-way radio latency; no post hoc correction is applied.

The backend-associated component showed much larger run-to-run variability than the local and gateway components, reinforcing the need for packet-level latency decomposition. Stage-specific sender, gateway, and database counters also show that database-visible continuity must be distinguished from successful local ESP-NOW delivery: exact stored PDR was 100% in the one-node runs, 99.28–99.88% in the three-node runs, and 96.75–97.05% in the five-node runs.

Future work should extend the same instrumentation to larger node populations, less controlled 2.4 GHz environments, event-driven and burst traffic, MQTT QoS 1/2, compiled gateway implementations, direct CPU/energy measurements, additional backend profiles, and hardware-assisted timestamping for applications requiring tighter absolute one-way timing accuracy.

## Figures and Tables

**Table 1:** Positioning of the present work with respect to representative literature on MQTT, ESP32 gateways, local 2.4 GHz communication, and IoT time synchronization.

Research Stream	Representative Studies	What Is Mainly Quantified or Demonstrated	Gap Addressed by This Work
MQTT broker and QoS performance	Borsatti et al. [10]; Bender et al. [11]; Mishra et al. [12]; Gaspar et al. [13]	Broker scalability, QoS behavior, resource consumption, payload size, client count, RTT, and end-to-end delivery latency.	Metrics are mostly broker-level or aggregate end-to-end values; embedded gateway firmware delay and local node-to-gateway timing are not decomposed.
Gateway, cloud, and cloud–edge MQTT placement	Kenitar et al. [14]; Ansyah et al. [15]; Tseng et al. [8]	Fog/cloud broker placement, cloud-provider performance, transient congestion, broker scheduling, and memory footprint.	The gateway is usually treated as a deployment element or broker-side component; internal ESP32/MicroPython gateway processing is not isolated.
IoT protocol comparison	Kang et al. [16]; Mishra and Guru [17]; Adriansyah et al. [18]	Comparison of MQTT with DDS, ZeroMQ, CoAP, HTTP, or ESP32-oriented communication alternatives using latency, throughput, energy, PDR, and overhead.	Protocol-level comparisons do not explain how delay is distributed across a complete ESP32 node–gateway–backend measurement path.
ESP-NOW and local 2.4 GHz ESP32 communication	Eridani et al. [19]; Becker et al. [20]	ESP-NOW range, payload constraints, latency, energy usage, barrier resistance, one-way delay distributions, jitter, and PDR.	The local link is evaluated largely as a standalone channel; its interaction with gateway scheduling, timestamping, and cloud ingestion is not characterized.
Embedded gateway runtime and scheduling	Plauska et al. [4]; Branch and Weinstock [23]; Ngo et al. [24]	Programming-language/runtime performance on ESP32, gateway throughput and loss under offered load, and RTOS task/core separation for time-critical radio and MQTT/network processing.	Existing studies show that runtime and scheduling architecture materially affect gateway behavior, but they do not decompose packet-level node-to-gateway, gateway-processing, and backend-ingestion timing for a cooperative MicroPython/uasyncio gateway.
ESP32 gateway feasibility and resource scalability	Gaspar et al. [21]; Serepas et al. [9]; Chang et al. [22]; Teslyuk et al. [28]	MicroPython rapid prototyping, MCU-class gateway feasibility, memory/resource use, device-count scaling, MQTT over TLS, local control, edge AI, buffering, and edge–cloud responsiveness.	These works demonstrate feasibility, resource trade-offs, and system-level responsiveness, but do not isolate the internal processing latency of a multi-node MicroPython sensing gateway.
Latency-sensitive MQTT and time synchronization	Patti et al. [29]; Testa et al. [30]; Chauhan and Gore [31]; Yiğitler et al. [32]; Mani et al. [33]	Prioritized MQTT, TSN integration, MQTT-based synchronization, clock discipline, clock drift, offset estimation, and synchronization accuracy.	These studies motivate latency-sensitive operation and timestamp uncertainty, but do not evaluate application-data latency components in a standard ESP32/MicroPython gateway pipeline.
Security, payload processing, and protocol interoperability	Gentile et al. [35]; Innamorati et al. [34]; Amirkhanova et al. [36]	TLS/SSL tunnels, cipher-suite effects, MQTT versions, MQTT–CoAP interoperability, and explicit ESP32-side serialization/encryption/encoding timing.	These studies quantify security and protocol-processing overheads, including firmware-side packet-preparation cost, but do not decompose multi-node gateway reception, cooperative scheduling, queueing, and backend-ingestion latency.
This work	ESP32 sensor nodes, ESP32–MicroPython gateway, ESP-NOW-based local link, MQTT/Telegraf/ InfluxDB backend	Two-way gateway-referenced timestamping, node-to-gateway timing metric, gateway-processing latency instrumentation, backend-forwarding timestamps, sequence continuity, and packet delivery behavior.	Provides diagnostic validation of a lightweight two-way timestamping method for application-level latency decomposition and reports repeated bridge-assisted one-, three-, and five-node periodic campaigns in a complete ESP32–MicroPython edge–cloud sensing pipeline.

**Table 2:** Summary of packet-level metrics used in the revised measurement campaign.

Metric	Full Name	Computation	Interpretation in This Work
$L_{n2g}$	Node-to-gateway delivery latency	$t_{\mathrm{gw},\mathrm{rx}}-t_{\mathrm{node}}$	Gateway-referenced application-level local delivery metric. It includes sender-side packet preparation after $t_{\mathrm{node}}$, ESP-NOW delivery, receiver timestamp placement, scheduler effects, and residual synchronization error; it is not interpreted as pure radio propagation delay.
$L_{\mathrm{gateway}}$	Gateway processing latency	$t_{\mathrm{gw},\mathrm{tx}}-t_{\mathrm{gw},\mathrm{rx}}$	Time from gateway packet reception to the MQTT publication call.
$L_{\mathrm{backend}}$	Gateway-to-backend ingestion latency	$t_{\mathrm{cloud}}-t_{\mathrm{gw},\mathrm{tx}}$	Delay after the gateway publication timestamp. In the bridge-assisted path, it includes local broker delivery, bridge forwarding to EMQX, Telegraf handling, and InfluxDB ingestion.
$L_{\mathrm{total}}$	End-to-end ingestion latency	$t_{\mathrm{cloud}}-t_{\mathrm{node}}$	Complete database-visible delay from the corrected sender timestamp to the backend ingestion timestamp.
$PDR_{\mathrm{stored}}$	Database-visible packet delivery ratio	Unique telemetry packets stored in InfluxDB divided by the exact sender-side transmission-attempt count reported for the measured run	Full-pipeline delivery metric. Sequence continuity is additionally reported as a diagnostic cross-check.
$d_{j}$	Two-way synchronization delay	$\left(t_{4,j}-t_{1,j}\right)-\left(t_{3,j}-t_{2,j}\right)$	Probe-level diagnostic used to reject delayed synchronization exchanges.
$w_{\theta }$	Offset-interval width	$\theta_{\mathrm{high}}-\theta_{\mathrm{low}}$	Width of the common admissible offset interval for the current three-probe cycle; reported as a synchronization-uncertainty diagnostic rather than a confidence interval.
Maximum gap	Maximum inter-packet gap	Largest time interval between consecutive stored packets within a node stream after duplicate removal	Indicator of transient delivery interruptions visible at the database level.
Throughput	Stored telemetry rate	Number of successfully stored telemetry packets per unit time	Practical database-visible telemetry rate for a given node-count scenario.

**Table 3:** Implementation and reproducibility details for the revised bridge-assisted measurement campaign.

Item	Value Used in the Revised Measurements
Sensor-node and gateway hardware	Custom sensor-node PCBs and gateway based on the Espressif ESP-WROOM-32 module (ESP32-D0WDQ6 SoC; Espressif Systems, Shanghai, China), with the gateway used as the local aggregation and timestamping unit.
Firmware environment	MicroPython v1.24.0 using uasyncio, ujson, and aioespnow.
Local node-to-gateway link	ESP-NOW-based 2.4 GHz communication.
Synchronization	Three-probe two-way gateway-referenced synchronization approximately every 20 s; telemetry and new sensor acquisition paused during active probes; current-cycle interval intersection used for the active offset estimate.
Synchronization probe policy	Three probes per cycle, 120 ms inter-probe gap, 700 ms response timeout, accepted delay 0–100 ms; unsuccessful cycles are retried while telemetry remains paused.
Gateway-to-local-broker transport	Plaintext MQTT over the controlled laboratory LAN; MQTT QoS 0; telemetry topic oasis/bottle.
Local bridge	Mosquitto v2.1.2 bridge on a Windows host, forwarding to EMQX Cloud over TLS.
Cloud and ingestion path	EMQX Cloud v5 broker, Raspberry Pi-based Telegraf v1.36.0 MQTT consumer, and InfluxDB Cloud Serverless (Storage Engine Version 3) time-series storage.
Telegraf latency profile	Output flush interval 1 s and flush jitter 0 s.
Traffic pattern	Periodic telemetry with nominal 1 s interval; the same startup de-phasing and bounded jitter policy is used in all node-count scenarios.
Gateway queue/publish policy	Queue capacity 500 packets; up to 4 MQTT publications per scheduling cycle; 5 ms idle sleep.
Evaluated scenarios	One, three, and five active sensor nodes; three independent 30 min repetitions per scenario.
Stored diagnostics	Sequence identifiers, exact sender transmission counters, run metadata, synchronization diagnostics, gateway queue/drop counters, MQTT error/requeue counters, publish-duration summaries, and free-memory diagnostics. The experimental measurement data and the analysis scripts used to process the reported results are provided as Supplementary Materials and are also available in the public GitHub repository at https://github.com/kashiakashia/esp32-edge-cloud-latency-data, accessed on 9 July 2026.

**Table 4:** Stage-specific reliability summary for the revised bridge-assisted campaign. Database PDR uses the exact sender-side TX denominator and complete deduplicated database sequence range for every run.

Scenario	Runs	Sender ACK	Gateway Drops	MQTT Publish Failures	Exact Database-Stored PDR
1 node	3	100%	0	0	100.000%, 100.000%, 100.000%
3 nodes	3	100%	0	0	99.281%, 99.880%, 99.397%
5 nodes	3	100%	0	0	96.900%, 96.747%, 97.047%

**Table 5:** Aggregate telemetry throughput for the three independent 30 min runs in each bridge-assisted scenario.

Scenario/Run	Duration [s]	Sender TX [pkt/s]	Database Stored [pkt/s]
1N RUN01	1800.030	0.7667	0.7667
1N RUN02	1800.320	0.7671	0.7671
1N RUN03	1800.210	0.7671	0.7671
3N RUN01	1801.150	2.3157	2.2991
3N RUN02	1801.350	2.3088	2.3060
3N RUN03	1801.220	2.3034	2.2896
5N RUN01	1801.120	3.8509	3.7316
5N RUN02	1800.840	3.8576	3.7321
5N RUN03	1800.840	3.8554	3.7416

**Table 6:** Revised bridge-assisted latency summary over three independent 30 min runs per scenario. Run-level means are reported as mean ± SD across the three run means; packet-level p95 values are shown descriptively.

Scenario	$L_{n2g}$ Mean [ms]	$L_{n2g}$ p95 [ms]	$L_{\mathrm{gateway}}$ Mean [ms]	$L_{\mathrm{backend}}$ Run-Mean [ms]	$L_{\mathrm{total}}$ Run-Mean [ms]
1 node	23.17 ± 0.13	28, 28, 28	13.31 ± 0.06	873.74 ± 267.61	910.22 ± 267.68
3 nodes	24.13 ± 0.12	33, 33, 33	13.40 ± 0.07	1108.84 ± 442.47	1146.38 ± 442.41
5 nodes	24.84 ± 0.47	38, 37, 38	13.46 ± 0.06	463.64 ± 109.49	501.94 ± 109.80

**Table 7:** Run-level synchronization-uncertainty diagnostics. Values are the median and p95 of the current-cycle admissible offset-interval width $w_{\theta }$ for each independent run.

Scenario	Run Medians of $w_{\theta }$ [ms]	Run p95 of $w_{\theta }$ [ms]
1 node	29, 29, 29	30, 30, 30
3 nodes	29, 29, 29	31, 31, 30
5 nodes	23, 28, 29	30, 30, 30

**Table 8:** Run-level effect-size summary for $L_{n2g}$. Differences are calculated from the three independent run-level values per scenario. Raw differences and percentage changes are emphasized because only three independent repetitions are available per condition.

Comparison	Run-Level Mean Difference [ms]	Mean Change [%]	Run-Level p95 Difference [ms]	p95 Change [%]
1N → 3N	+0.97	+4.2	+5.00	+17.9
1N → 5N	+1.68	+7.2	+9.67	+34.5
3N → 5N	+0.71	+3.0	+4.67	+14.1

**Table 9:** Independent oscilloscope validation of the software-derived node-to-gateway latency. The final row reports the total number of analyzed packets, the mean ± SD across the three run means for the Rigol and software mean values, the pooled software median and p95, the mean ± SD of the run-level software–hardware differences, and the pooled median synchronization interval width.

Run	Packets	Rigol Mean [ms]	SW Mean [ms]	SW Median [ms]	SW p95 [ms]	$\Delta_{\mathrm{SW}-\mathrm{HW}}$ [ms]	Median $w_{\theta }$ [ms]
Validation 1	690	15.899	26.167	25	40	+10.268	28
Validation 2	679	15.100	27.328	24	42	+12.228	29
Validation 3	686	12.638	26.539	23	42	+13.901	29
Across runs	2055	14.546 ± 1.700	26.678 ± 0.593	24	42	+12.132 ± 1.819	29

**Table 10:** Contextual comparison of selected latency and timing values reported in related studies. Values are not directly comparable because the studies evaluate different protocol layers, hardware platforms, and timing definitions.

Study	Evaluated Layer/Scenario	Reported Quantitative Values	Relevance to This Work
Becker et al. [20]	ESP-NOW one-way delay and PDR in outdoor field experiments	Good links: average delay of approximately 2.78 ms to 3.46 ms with PDR close to 100%; degraded links showed substantially lower PDR and larger delay variability.	Shows that delivered-packet ESP-NOW link delay alone is expected to be far below hundreds of milliseconds under good conditions; therefore, application-level $L_{n2g}$ must include software and timestamping effects.
Eridani et al. [19]	Comparison of ESP-NOW, Wi-Fi, and Bluetooth on ESP32-class devices	ESP-NOW is reported as a low-overhead local communication option with favorable latency and energy properties compared with alternative short-range configurations.	Supports the selection of ESP-NOW for the local node-to-gateway segment, but does not include cloud forwarding or MicroPython gateway scheduling.
Mani et al. [33]	Clock synchronization in IoT prototyping platforms	Reported synchronization inaccuracies of approximately 700 ms to 1600 ms and clock drift up to approximately 600 ms over short periods; the proposed SPoT method maintained accuracy around 15 ms.	Motivates explicit synchronization diagnostics and the use of a two-way offset-estimation procedure rather than relying on simple one-way timestamp broadcasts.
Gaspar et al. [13]	MQTT stress testing under different QoS levels, payloads, client counts, and broker settings	The study evaluates RTT, packet loss, and end-to-end publishing delay as functions of QoS, payload, queue size, broker resources, and number of clients.	Useful context for MQTT reliability–latency trade-offs, but not directly comparable with gateway-processing latency because it focuses on MQTT/broker behavior.
This work	ESP32 sensor node, ESP32–MicroPython gateway, ESP-NOW local link, local Mosquitto bridge, EMQX/Telegraf/InfluxDB backend	Bridge-assisted one-, three-, and five-node campaigns: run-level mean $L_{n2g}$ 23.17 ± 0.13 ms, 24.13 ± 0.12 ms, and 24.84 ± 0.47 ms; packet-level p95 28 ms, 33 ms, and approximately 37–38 ms; run-level mean $L_{\mathrm{gateway}}$ 13.31 ± 0.06 ms, 13.40 ± 0.07 ms, and 13.46 ± 0.06 ms. Independent hardware validation showed a software-minus-hardware run-mean difference of +12.132 ± 1.819 ms.	Provides packet-level decomposition in a MicroPython ESP32 edge–cloud pipeline, independently constrains the interpretation of the software timestamping metric, and reports repeated bridge-assisted behavior from one to five active nodes without claiming a quantitative direct-versus-bridge improvement.

## Data Availability

The experimental data supporting the findings of this study are openly available in the public GitHub repository: https://github.com/kashiakashia/esp32-edge-cloud-latency-data, accessed on 9 July 2026.

## Supplementary Materials

The following supporting information can be downloaded at https://www.mdpi.com/article/10.3390/s26175555/s1.

## Abbreviations

The following abbreviations are used in this manuscript:

BLE	Bluetooth Low Energy
CoAP	Constrained Application Protocol
CSV	Comma-separated values
DDS	Data Distribution Service
ESP32	Espressif Systems 32-bit microcontroller platform
IIoT	Industrial Internet of Things
IoT	Internet of Things
MQTT	Message Queuing Telemetry Transport
NTP	Network Time Protocol
PDR	Database-visible packet delivery ratio
QoS	Quality of service
RTT	Round-trip time
TLS	Transport Layer Security
TSN	Time-Sensitive Networking

## Appendix A. Run-Level Campaign Statistics

**Table A1:** Run-level latency statistics for the revised bridge-assisted periodic campaign.

Run	$L_{n2g}$ Mean [ms]	$L_{n2g}$ Median/p95 [ms]	$L_{\mathrm{gateway}}$ Mean [ms]	$L_{\mathrm{backend}}$ Mean [ms]	$L_{\mathrm{total}}$ Mean [ms]
1N-RUN01	23.02	23/28	13.38	564.75	601.15
1N-RUN02	23.24	23/28	13.28	1025.70	1062.22
1N-RUN03	23.24	23/28	13.28	1030.78	1067.30
3N-RUN01	24.08	23/33	13.33	1572.98	1610.39
3N-RUN02	24.27	23/33	13.43	1061.74	1099.44
3N-RUN03	24.05	23/33	13.46	691.81	729.32
5N-RUN01	25.03	23/38	13.52	407.38	445.93
5N-RUN02	25.20	23/37	13.43	589.82	628.45
5N-RUN03	24.31	23/38	13.42	393.72	431.44

**Table A2:** Run-level stage-specific reliability for the revised bridge-assisted periodic campaign. Database values are exact full-run stored PDR values based on sender TX denominators and complete deduplicated database sequence ranges.

Run	Sender ACK	Gateway Drops	MQTT Publish Failures	Database Metric [%]	Interpretation
1N-RUN01	100%	0	0	100.000	Exact full-run stored PDR.
1N-RUN02	100%	0	0	100.000	Exact full-run stored PDR.
1N-RUN03	100%	0	0	100.000	Exact full-run stored PDR.
3N-RUN01	100%	0	0	99.281	Exact full-run stored PDR.
3N-RUN02	100%	0	0	99.880	Exact full-run stored PDR; one duplicate record observed.
3N-RUN03	100%	0	0	99.397	Exact full-run stored PDR.
5N-RUN01	100%	0	0	96.900	Exact full-run stored PDR.
5N-RUN02	100%	0	0	96.747	Exact full-run stored PDR; 6947/6947 sender ACK, no send errors, no gateway drops, no MQTT publication failures.
5N-RUN03	100%	0	0	97.047	Exact full-run stored PDR; 6943/6943 sender ACK, no send errors, no gateway drops, no MQTT publication failures.
